# WNT3a Signaling Inhibits Aromatase Expression in Breast Adipose Fibroblasts—A Possible Mechanism Supporting the Loss of Estrogen Responsiveness of Triple-Negative Breast Cancers

**DOI:** 10.3390/ijms24054654

**Published:** 2023-02-28

**Authors:** Alexander Kaiser, Gabriele Eiselt, Joachim Bechler, Otmar Huber, Martin Schmidt

**Affiliations:** 1 Institute for Biochemistry II, Jena University Hospital, Friedrich Schiller University, 07743 Jena, Germany; 2 Department of Gynecology and Obstetrics, Robert-Koch-Hospital, 99510 Apolda, Germany

**Keywords:** aromatase, breast cancer, breast adipose fibroblast, Wnt signaling, LEF-1 (lymphoid enhancer binding factor 1), TCF-4 (T-cell factor 4), β-catenin, gene regulation

## Abstract

Estrogen-dependent breast cancers rely on a constant supply of estrogens and expression of estrogen receptors. Local biosynthesis, by aromatase in breast adipose fibroblasts (BAFs), is their most important source for estrogens. Triple-negative breast cancers (TNBC) rely on other growth-promoting signals, including those from the Wnt pathway. In this study, we explored the hypothesis that Wnt signaling alters the proliferation of BAFs, and is involved in regulation of aromatase expression in BAFs. Conditioned medium (CM) from TNBC cells and WNT3a consistently increased BAF growth, and reduced aromatase activity up to 90%, by suppression of the aromatase promoter I.3/II region. Database searches identified three putative Wnt-responsive elements (WREs) in the aromatase promoter I.3/II. In luciferase reporter gene assays, promoter I.3/II activity was inhibited by overexpression of full-length T-cell factor (TCF)-4 in 3T3-L1 preadipocytes, which served as a model for BAFs. Full-length lymphoid enhancer-binding factor (LEF)-1 increased the transcriptional activity. However, TCF-4 binding to WRE1 in the aromatase promoter, was lost after WNT3a stimulation in immunoprecipitation-based in vitro DNA-binding assays, and in chromatin immunoprecipitation (ChIP). In vitro DNA-binding assays, ChIP, and Western blotting revealed a WNT3a-dependent switch of nuclear LEF-1 isoforms towards a truncated variant, whereas β-catenin levels remained unchanged. This LEF-1 variant revealed dominant negative properties, and most likely recruited enzymes involved in heterochromatin formation. In addition, WNT3a induced the replacement of TCF-4 by the truncated LEF-1 variant, on WRE1 of the aromatase promoter I.3/II. The mechanism described here may be responsible for the loss of aromatase expression predominantly associated with TNBC. Tumors with (strong) expression of Wnt ligands actively suppress aromatase expression in BAFs. Consequently a reduced estrogen supply could favor the growth of estrogen-independent tumor cells, which consequently would make estrogen receptors dispensable. In summary, canonical Wnt signaling within (cancerous) breast tissue may be a major factor controlling local estrogen synthesis and action.

## 1. Introduction

In postmenopausal women, the production of estrogens is located mainly in extragonadal tissue, preferentially in breast adipose fibroblasts (BAFs) [1]. Estrogen synthesis from androgens depends on three consecutive oxidation steps, which are catalyzed by the cytochrome P-450 enzyme aromatase, encoded by the *CYP19A1* gene [2,3]. Estrogens are the most important female sex hormones, but they can also act as important growth factors in breast cancers. BAFs in the desmoplastic area, in the environment of estrogen receptor (ER)-positive breast tumors, increasingly express the aromatase enzyme and synthesize estrogens [1]. Mechanistically, the expression of aromatase in BAFs, which mainly comprise preadipocytes, is regulated at the transcriptional level. Different tissue-specific aromatase promoters have been identified as regulating expression of coding exons II-X [4]. In this context, specific signaling factors from ER-positive breast cancer cells have been shown to activate aromatase promoters I.3 and II in BAFs. Thus, promoters I.3 and II are responsible for 80–90% of aromatase expression in the tumor environment [1]. Consequently, aromatase inhibition has emerged as an efficient therapy for ER-positive breast cancers [5].

On the other hand, long-term prognosis and therapeutic options are much poorer in triple-negative breast cancers (TNBC), which by definition do not express ERα and progesterone receptor (PR), and do not overexpress the receptor tyrosine-protein kinase erbB-2 (HER2). A key role in these tumors is attributed to the Wnt signaling pathway [6,7,8,9]. Wnt signaling is essential for many developmental processes, whereas deregulation of pathway-related factors contributes to oncogenesis (e.g., in colorectal cancers [10]). Nineteen different Wnt ligands induce β-catenin-dependent canonical (e.g., WNT1, WNT3A and WNT8), or β-catenin-independent noncanonical (e.g., WNT5A and WNT11), signaling [6]. In canonical signaling, high mobility group (HMG)-box transcription factors of the TCF/LEF family (TCF: T-cell factor; LEF: lymphoid enhancer factor) are the main binding partners for β-catenin in nuclear gene regulation. A high diversity of TCF/LEFs variants mediates a broad spectrum of activating and inhibitory functions. For example, promoter switching to more 3′-regions in the *TCF/LEF* genes results in isoforms without β-catenin binding domains (dnLEF-1, dnTCF-4) acting as dominant-negative proteins [11,12,13,14].

Active Wnt signaling is not only associated with the normal development of the mammary gland, but also with the loss of ERα, or a lack of HER2 amplification in TNBC [15,16,17]. In addition, active Wnt signaling also contributes to tumor–stroma interactions in TNBC [18]. However, there is little knowledge about the mechanism, if any, of aromatase regulation in these tumors. Interestingly, a recent study provides evidence for aromatase expression in only a limited number of TNBC [19]. Furthermore, there is evidence that canonical Wnt signaling inhibits follicle stimulating, hormone-mediated aromatase expression in primary cultures of rat granulosa cells [20].

Therefore, we set out to elucidate the potential relationship of Wnt signaling and aromatase expression in BAFs. Using conditioned media from the WNT3a-secreting TNBC cell line MDA-MB231 [18], and from WNT3a-overexpressing L-M(TK-) cells, we provide evidence for, growth stimulatory and aromatase suppressing activity of Wnt signaling in BAFs. Furthermore, we identified Wnt response elements in the breast cancer relevant aromatase promoter I.3/II region, and identified a switch in promoter occupancy from TCF-4 to a LEF-1 variant, which appears to be involved in the WNT3a induced suppression of aromatase in BAFs.

## 2. Results

### 2.1. Breast Cancer Cell- and WNT3a-Conditioned Media Potentiate BAF Growth

**Figure 1:**
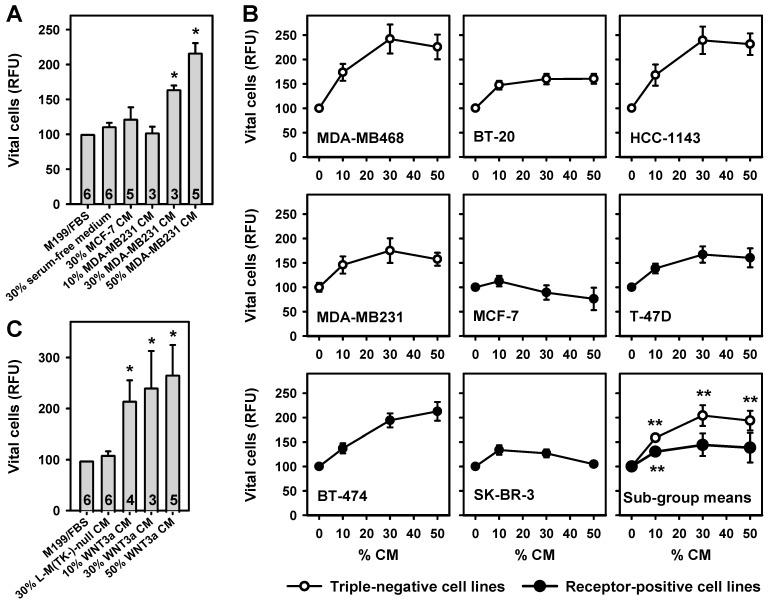
Breast cancer cell line conditioned medium (CM) and WNT3a CM potentiate breast adipose fibroblast (BAF) proliferation. Starting two days after seeding, BAFs were incubated for 8 days with various concentrations of CM. Media were changed every other day. Vital cells were assayed with the fluorescein diacetate (FDA) assay. (**A**) BAFs were cultured in M199 medium, without or with different volume percentages of serum-free CM from MDA-MB231 cells, MCF-7 cells, or with 30% serum-free media alone. MCF-7 CM caused no significant changes in vital cells (parameter for vital cells: RFU, relative fluorescence units). MDA-MB231 CM induced a dose-dependent increase in vital cells. Numbers indicate biological replicates tested in triplicate. (**B**) BAFs were grown in the presence of 50% M199/FBS and various concentrations of serum-free CM from the indicated cell lines (the balance was filled with serum-free medium). n = 2 experiments tested in quadruplicate. Subgroup mean values were calculated from the mean values obtained for the triple-negative and receptor-positive cell lines, respectively. (**C**) WNT3a CM induced a concentration-dependent increase in vital cells, whereas 30% serum-free CM from L-M(TK-)-null cells, not expressing WNT3a, had no such effect. All values represent means ± SEM, and were normalized to 100% M199/fetal bovine serum (FBS) (*, *p* < 0.05; **, *p* < 0.005; versus M199/FBS or 0% CM, respectively).

Proliferation of breast adipose fibroblasts (BAFs), in response to conditioned medium (CM), was measured using the fluorescein diacetate (FDA) viability assay. In pilot experiments, a significant and dose-dependent increase in BAF growth was detectable when cells were cultured in the presence of serum-free CM from ERα-negative MDA-MB231 breast cancer cells (more than 2-fold increase at 50% CM, Figure 1A). This WNT3a-expressing cell line served as a model for TNBC [18]. In contrast, conditioned medium from the ERα-positive MCF-7 breast cancer cell line did not induce significant proliferation (Figure 1A).

**Figure A1:**
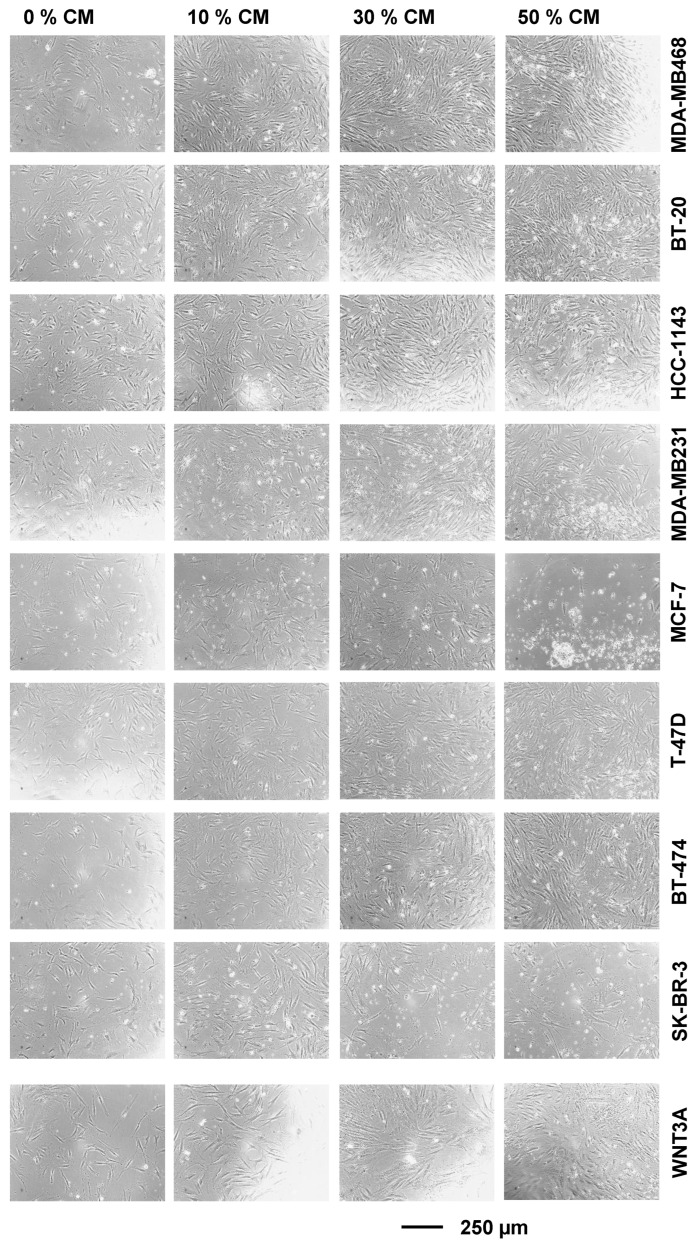
Breast cancer cell line CM and WNT3a CM potentiate BAF proliferation. Microscopic images of representative wells from an experiment subsequently used for the proliferation assay (for details see legend to Figure 1B,C).

To clarify whether secretion of factors promoting the proliferation of BAFs is mainly a property of TNBC cells, the growth promoting activities of four TNBC cell lines, and four lines expressing various combinations of the respective receptors, were compared (Table 1). Whereas all TNBC cell line CM significantly stimulated BAF growth, the effects of CM from receptor-positive cell lines varied considerably (Figure 1B). These results are in accordance with the microscopic assessment of the cultures immediately before the FDA assay was started (Appendix A, Figure A1). When the mean effects of the subgroups were compared, TNBC cell lines solidly stimulated BAF growth over a wide range of CM concentrations, whereas the receptor-positive cell lines only marginally stimulated BAF growth (Figure 1B). We tested additional features of the cell lines for their influence on BAF proliferation: source of cell line (primary breast cancer, pleural effusion), tumor type (adenocarcinoma, invasive ductal carcinoma, ductal carcinoma), and (over)expression or mutation of cellular tumor antigen p53 (*TP53*). None of these classifications is associated with stronger or weaker promotion of BAF growth.

To test whether Wnt signaling may contribute to the growth promoting effect of tumor cell CM, WNT3a CM, obtained from L-M(TK-)WNT3a cells, was used [24]. WNT3a was chosen because it is a prototypical ligand stimulating the canonical Wnt signaling pathway [6], and because this producer cell line provides high titers of bioactive ligand. Indeed, WNT3a CM also dose-dependently induced significant BAF proliferation, resulting in a more than 2.5-fold stimulation with 50% WNT3a CM (Figure 1C). Microscopy confirmed these findings (Appendix A, Figure A1).

### 2.2. Expression of WNT1 and WNT3A in Breast Cancer Cell Lines

**Figure A2:**
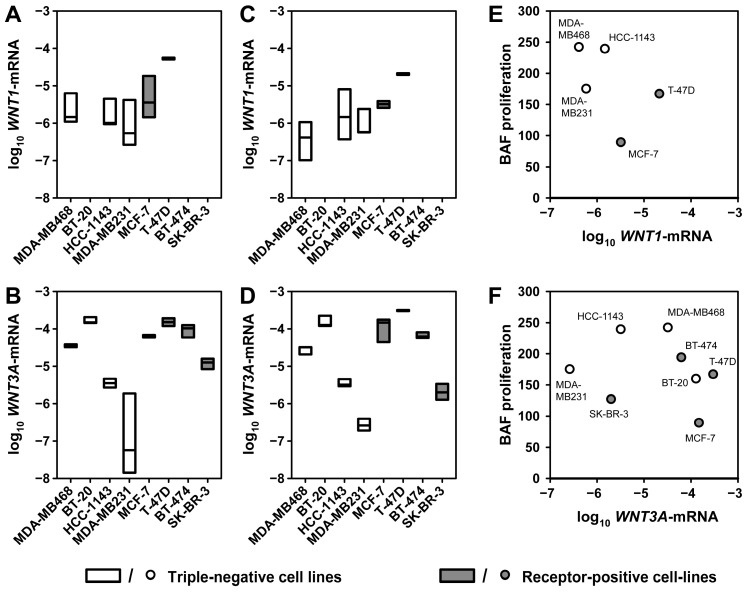
*WNT1* and *WNT3A* expression in breast cancer cell lines. RNA was isolated from cells used for production of CM under FBS containing or serum-free conditions, respectively, for three days. Expression levels were quantified by qPCR and are normalized to *GAPDH* expression. (**A**) *WNT1* and (**B**) *WNT3A* expression in the presence of FBS. (**C**) *WNT1* and (**D**) *WNT3A* expression in the absence of serum. The boxes indicate median and range for three independent experiments, absence of box indicates lack of quantifiable expression. (**E**) *WNT1* and (**F**) *WNT3A* expression in the absence of serum were correlated with the proliferation rates of BAFs treated with 30% CM (indicated as vital cells in Figure 1B).

To verify the expression of Wnt isoforms, RNA was isolated from all cell lines after three days of media conditioning—both in the presence and absence of serum. *WNT1* mRNA expression was detectable in MDA-MB468, HCC-1143, MDA-MB231, MCF-7, and T-47D cells kept in fetal bovine serum (FBS)-containing media (Appendix A, Figure A2A). *WNT3A* mRNA expression was detectable in all investigated cell lines in the presence of FBS (Appendix A, Figure A2B). The expression patterns of *WNT1* and *WNT3A* were only marginally altered under serum-free conditions (Appendix A, Figure A2C,D), indicating constitutive expression of these genes in the cell lines. Correlation analysis of the BAF proliferation stimulating activity of CM with WNT gene expression yielded no positive association of *WNT1* or *WNT3A* expression with BAF growth (Appendix A, Figure A2E,F). This indicates that both, WNT1 and WNT3a, may contribute to the growth promotion, but are not the sole factors in the CM doing this.

### 2.3. Aromatase Activity and Expression in BAFs Treated with MDA-MB231 CM or WNT3a CM

**Figure 2:**
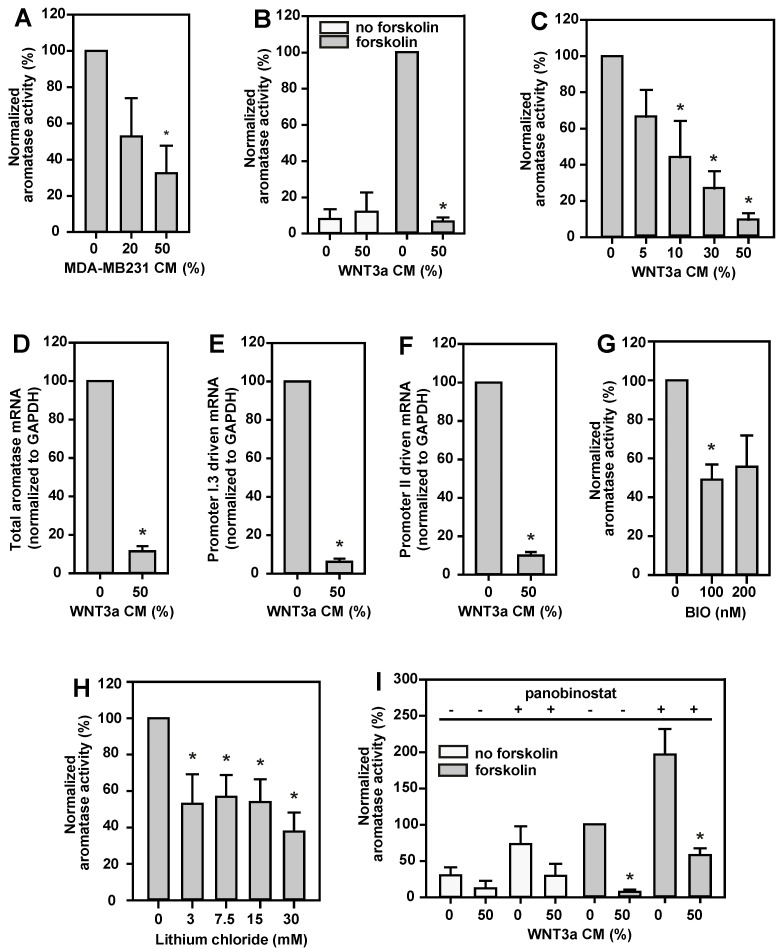
Aromatase in BAFs is inhibited by MDA-MB231 CM, canonical Wnt signaling, or active HDACs. BAFs were incubated for 24 h in serum-free medium supplemented with 10 µM forskolin for aromatase induction (grey bars), or vehicle (no forskolin, white bars). Different volume percentages of MDA-MB231 CM, WNT3a CM, or different concentrations of glycogen synthase kinase-3β (GSK-3β) inhibitors (BIO, lithium chloride), or the histone deacetylase (HDAC) inhibitor panobinostat (50 nM) were applied throughout the incubation time, as indicated. For measurement of aromatase activity, ^3^H-labeled androstenedione was added 6 h before termination of the incubation time. Cell lysates for mRNA analyses were prepared after 24 h of treatment with forskolin, in the absence or presence of 50% (*v*/*v*) WNT3a CM. (**A**) MDA-MB231 CM inhibited aromatase activity dose-dependently. (**B**) Basal aromatase activity was not affected by WNT3a CM, however, forskolin stimulated aromatase activity was reduced by more than 90% in the presence of 50% (*v*/*v*) WNT3a CM. (**C**) This inhibitory effect of WNT3a CM was dose-dependent. (**D**) Consistently, aromatase mRNA expression was inhibited upon treatment of BAFs with WNT3a CM. (**E**,**F**) WNT3a CM similarly suppressed transcription via promoters I.3 and II, respectively, on RNA level. The effects of the GSK-3β inhibitors BIO (**G**) and lithium chloride (**H**) confirm the involvement of canonical Wnt signaling in the mechanism of WNT3a-triggered inhibition of aromatase activity. (**I**) HDAC inhibition by panobinostat markedly increased aromatase activity, and WNT3a CM significantly inhibited aromatase activity in panobinostat-treated BAFs. Data are normalized to values from cells treated with forskolin alone, and are presented as means ± SEM of 3 (in **A**,**C**,**G**–**I**) or 4 (in **B**,**D**–**F**) independent experiments. Significant differences, as compared to values for the respective controls treated with forskolin, were identified by Student’s *t*-test (*, *p* < 0.05) or (in **B**,**D**–**F**) the Mann–Whitney rank sum test (*, *p* < 0.05).

Aromatase activity in BAFs was stimulated with forskolin, in order to mimic the tumor–stroma situation in the vicinity of breast tumors [1]. Under these conditions, the aromatase activity of BAFs was inhibited dose-dependently by MDA-MB231 CM, to less than 40% of controls (Figure 2A). WNT3a CM (50%) had no major effect on basal aromatase activity in BAFs. By contrast, it revealed a strong inhibition (90%) of forskolin stimulated aromatase activity (Figure 2B). This effect was strongly dose-dependent (Figure 2C).

WNT3a CM inhibited full-length aromatase mRNA expression by up to 90% in BAFs (Figure 2D), indicating that WNT3a exerts its effect on aromatase gene expression at the transcriptional level. Furthermore, the expression levels of aromatase mRNA transcripts with 5’-ends, typical for transcription controlled by promoters I.3 or II, decreased similarly to that of the full-length aromatase gene expression level (Figure 2E,F). This means that WNT3a massively antagonizes the breast cancer relevant mechanism of aromatase induction in BAFs, here experimentally mimicked by forskolin stimulation.

### 2.4. Aromatase Activity Is Inhibited by Canonical Wnt Signaling and Histone Deacetylases

Canonical Wnt signaling is activated by inhibition of glycogen synthase kinase-3β (GSK-3β). Indeed, inhibition of GSK-3β by BIO (Figure 2G) or lithium chloride (Figure 2H) dose-dependently reduced aromatase activity to less than 50% and 40%, respectively. Toxic effects of the inhibitors were excluded by FDA tests. In summary, this indicates an involvement of the canonical Wnt signaling pathway, and the β-catenin/TCF transcription complex, in inhibition of aromatase expression in BAFs.

The activity of the β-catenin/TCF transcription complex is modulated by multiple interaction partners associated with epigenetic regulation, including histone acetyltransferases (HATs) and histone deacetylases (HDACs) [25,26]. In this context we observed that aromatase activity in BAFs increased significantly after nonselective HDAC inhibition by panobinostat under breast cancer mimicking conditions (forskolin stimulation, Figure 2I). A less pronounced effect was seen under basal conditions (without forskolin). Therefore, HDACs must be involved in promoter I.3- and II-dependent aromatase expression. Importantly, WNT3a stimulation led to an inhibition of aromatase activity in BAFs—even in the presence of an HDAC inhibitor, i.e., in a state of de-repression of transcription (Figure 2I). Thus, both WNT3a-treatment and active HDACs, resulted in an inhibition of aromatase activity.

### 2.5. Identification of Putative Wnt Response Elements in the Aromatase Promoter I.3/II Sequence

**Figure 3:**
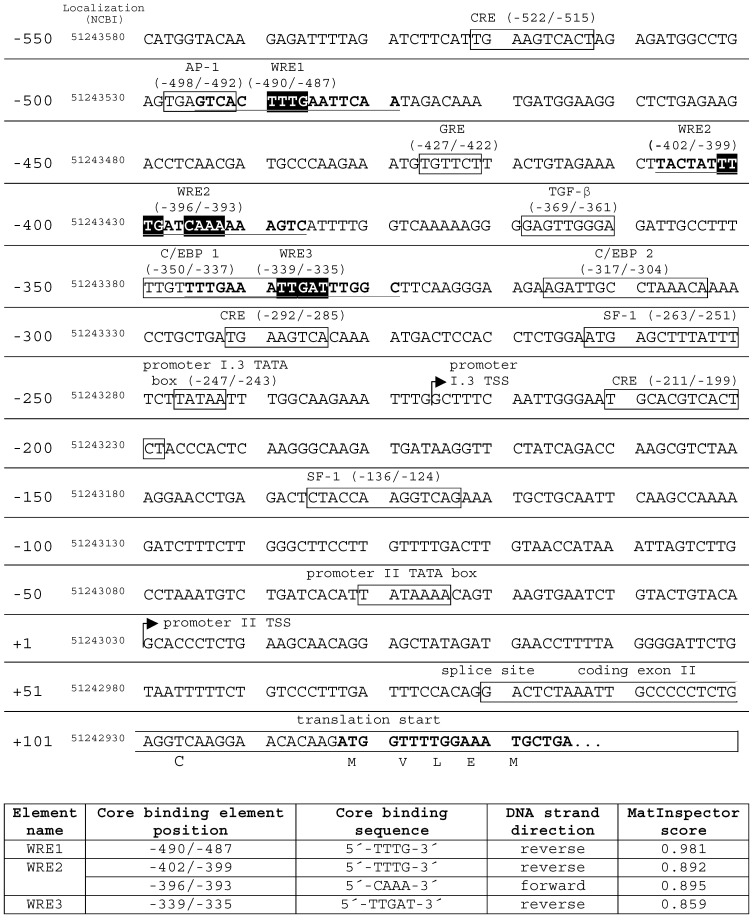
Transcription factor binding sites and Wnt response elements (WRE) in the aromatase promoter I.3/II. MatInspector (Genomatix) database searches revealed three putative WREs in the aromatase promoter I.3/II region (GenBank Nc_000015.10 chromosome 15 reference GRCh38 primary assembly). The core binding element positions are indicated with respect to the promoter II transcriptional start site (TSS). In addition, their genomic localization is given. WRE core binding elements are highlighted in black; full-length WRE sequences are in bold type and underlined. WRE2 combined two core binding sequences in forward and reverse direction, respectively. WRE search results (lower table) were evaluated by MatInspector’s specific matrix similarity score. The higher the score, the higher the sequence identity with the ideal WRE sequence (maximum score 1). The promoter elements in boxes are derived from a review of Chen et al. [1].

Putative target DNA elements of (canonical) Wnt signaling in aromatase promoter I.3/II were identified in silico, by MatInspector (Genomatix, Munich, Germany) database searches, revealing three Wnt response elements (WRE1, WRE2, WRE3) up to 495 bp upstream of the promoter II transcriptional start site (Figure 3). The sequence matching best was WRE1 (position −495/−480; MatInspector score: 0.981), and is located directly downstream of an AP-1 element. WRE3 (position −346/−330) overlapped with a C/EBP1 element. Both the AP-1 and C/EBP1 elements are known to be involved in activation of the aromatase promoter I.3/II region [1]. WRE2 presents as a combination of two binding sites (position −408/−387), and is located between WREs 1 and 3.

### 2.6. Evaluation of Putative WREs in the Aromatase Promoter I.3/II Region In Vitro

**Figure 4:**
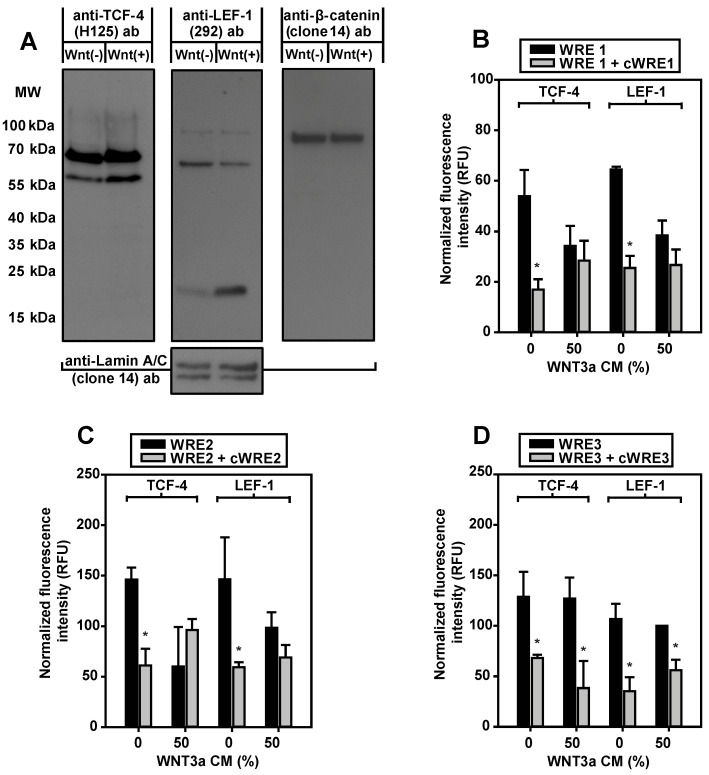
Expression and in vitro WRE-binding of Wnt signaling proteins. (**A**) 3T3-L1 preadipocytes were incubated overnight in serum-free medium with 10 µM forskolin in the absence (Wnt(−)) or in the presence of 50% WNT3a CM (Wnt(+)). For Western blotting, every lane contained 100 µg soluble nuclear extract. Membranes were (re)probed from left to right with the antibodies (ab) indicated. Comparable protein loading was tested by anti-Lamin A/C antibody. MW, molecular weight. One of two experiments is shown. (**B**–**D**) For each condition, 50 µg soluble nuclear extracts from 3T3-L1 cells were subjected to immunoprecipitation, with either anti-T-cell factor-4 (TCF-4) (L40C3) or anti- lymphoid enhancer factor-1 (LEF-1) (2D12) antibody. The precipitates were incubated with WRE-specific fluorescent double-stranded oligonucleotides (WRE1, WRE2, WRE3). Binding of labeled WRE-oligonucleotides was suppressed by an excess of the corresponding nonfluorescent competitor oligonucleotide (cWRE1, cWRE2, cWRE3). Immunoprecipitates from WNT3a-stimulated cells showed no specific binding to WRE1 or WRE2. Means ± SE; n = 3; Student’s *t*-test (*, *p* < 0.05).

As an established model for studies on the regulation of aromatase [27], and due to their unlimited availability, murine 3T3-L1 preadipocytes were used for detailed evaluation of the putative WREs. In nuclear extracts from 3T3-L1 cells, mediators of Wnt signaling, TCF-4, LEF-1 and β-catenin were detectable by Western blotting (Figure 4A). For TCF-4, the smaller isoform (apparent MW 60 kDa) increased after WNT3a stimulation. An even more pronounced change of isoform expression during WNT3a stimulation was found for LEF-1. The larger isoform markedly decreased in intensity, whereas expression of a short variant of LEF-1 increased strikingly.

To elucidate whether native LEF-1 and TCF-4 were able to bind to the putative WREs identified in the aromatase promoter I.3/II region in vitro, an immunoprecipitation-based oligonucleotide binding assay was established. In contrast to the Western blot experiments, epitopes of antibodies used for immunoprecipitation were located within the N-terminal regions, to avoid interference of antibody binding with DNA-binding. TCF-4 and LEF-1 immunoprecipitates bound all three WREs (Figure 4B–D). The specific DNA-binding was inhibited by nonfluorescent WRE competitor oligonucleotides, with the same sequences. Remarkably, WNT3a treatment inhibited specific DNA-binding of LEF-1 and TCF-4 immunoprecipitates to WRE1 and WRE2, whereas this effect was not detectable with WRE3. This indicates that, at least WREs 1 and 2 are responsive to Wnt signaling.

### 2.7. WNT3a Treatment Triggers TCF-4 Replacement by LEF-1 on WRE1 of the Aromatase Promoter I.3/II Region in BAFs

**Figure 5:**
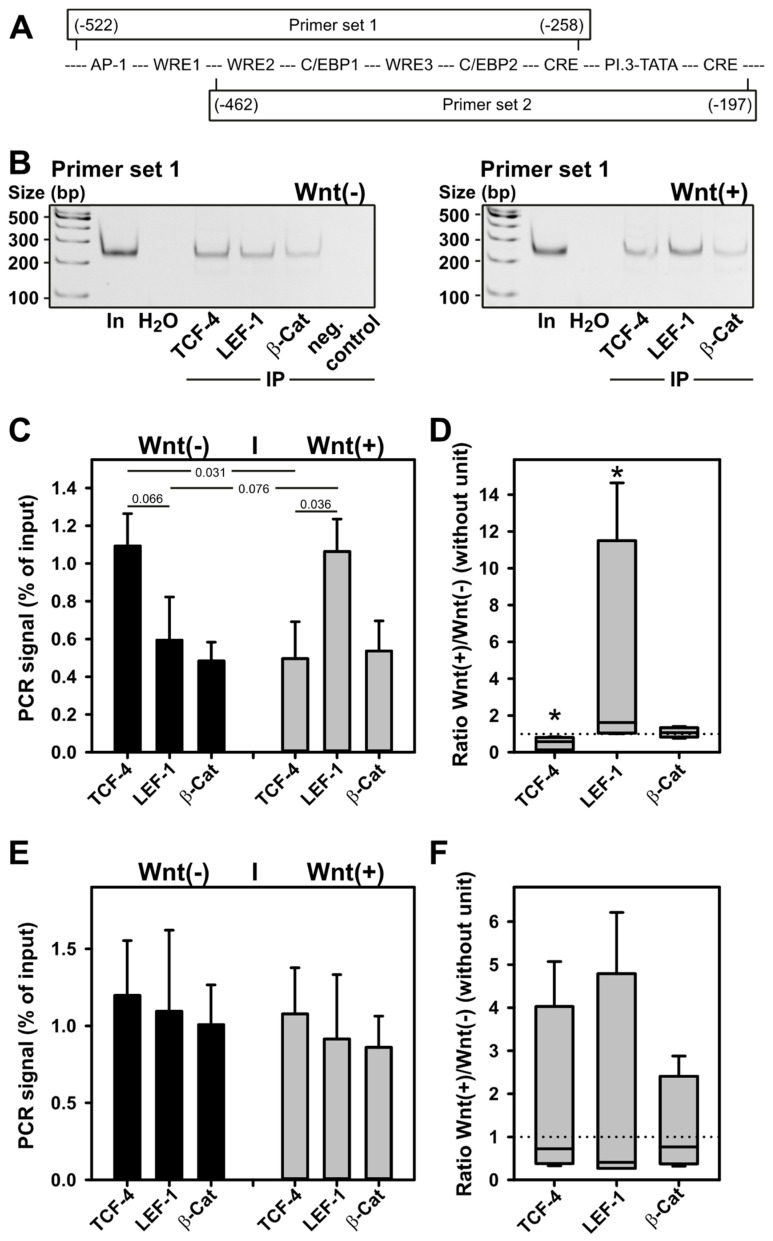
LEF-1 replaces TCF-4 on WRE1 of the aromatase promoter in BAFs upon WNT3a treatment. BAFs were incubated for 24 h in serum-free medium with 10 µM forskolin ±50% WNT3a CM (indicated as Wnt(−) and Wnt(+), respectively). ChIP was performed as described in Methods. Antibodies used for immunoprecipitation were anti-TCF-4 (H-125) (TCF-4), anti-LEF-1 292 (LEF-1), or anti-β-catenin (clone 14) (β-Cat). (**A**) Two primer sets were used for subsequent PCR: primer set 1 defines an amplicon, which includes all WREs in the aromatase promoter I.3/II region, whereas primer set 2 amplifies a region containing only WRE2 and WRE3. Boxes representing amplicons: numbers in parentheses indicate position in relation to the promoter II transcriptional start site; middle line indicates positions of identified and putative cis-elements in the promoter region (for details see Figure 3). (**B**) Representative polyacrylamide gels from one PCR with primer set 1. In: 1:50 diluted DNA input; H_2_O: water used as PCR control without template; neg. control: Protein G Sepharose alone used for immunoprecipitation (IP). For quantitation, band intensities obtained from ChIP reactions were expressed as %-values of input signal: (**C**) Means ± SEM obtained with BAFs from (n = 4) donors using primer set 1. Student’s *t*-test was used to identify differences between groups (horizontal bars, numbers indicate *p*-values). (**D**) Ratios of band intensities obtained for WNT3a-treated and untreated BAFs, using primer set 1, were calculated for each antibody target examined. No response to treatment equals a ratio of 1 (dotted line). Differences to a no-response situation were identified with the Mann–Whitney rank sum test (n = 4; *, *p* < 0.05). (**E**) Means ± SEM obtained with BAFs from (n = 4) donors using primer set 2. (**F**) Ratios of WNT3a responses using primer set 2.

The evidence obtained so far indicated a possible role of WRE-bound transcription factors of the TCF-4/LEF-1 family in the WNT3a-induced inhibition of transcription from the aromatase promoter I.3/II region. To analyze their role in vivo, chromatin immunoprecipitation (ChIP) experiments were performed with forskolin stimulated BAFs, in the presence or absence of WNT3a CM. The antibodies used for the immunoprecipitations were those used for Western blotting, thus allowing differentiation between the large and small variants of TCF-4 and LEF-1, respectively. Because WRE1 is almost identical to the WRE-consensus sequence (see Figure 3), primer sets for polymerase chain-reaction (PCR) were constructed, to differentiate WRE1-mediated binding of proteins from binding to the other WREs (Figure 5A).

With primer set 1, TCF-4 binding to the WRE region of aromatase promoter I.3/II significantly decreased upon WNT3a stimulation (Figure 5B,C). In contrast, LEF-1 binding tended to increase under WNT3a stimulation. When the ratios of band intensities obtained for WNT3a-treated and -untreated BAFs were calculated for each antibody target examined, both the reduction in TCF-4 binding, and the increase in LEF-1 binding, triggered by WNT3a were statistically significant (Figure 5D). When transcription factor binding was analyzed analogously with primer set 2 lacking WRE1, no effect of WNT3a treatment was observed (Figure 5E,F). For β-catenin binding, no effect of WNT3a treatment was detectable, using either primer set. In summary, TCF-4 and LEF-1 bind to WREs in aromatase promoter I.3/II region in vivo. On WRE1 TCF-4 binding dominates under nonstimulated conditions, whereas LEF-1 binding dominates after WNT3a stimulation.

### 2.8. Functional Consequences of TCF-4 or LEF-1 Binding to WREs in the Aromatase Promoter I.3/II Region

**Figure 6:**
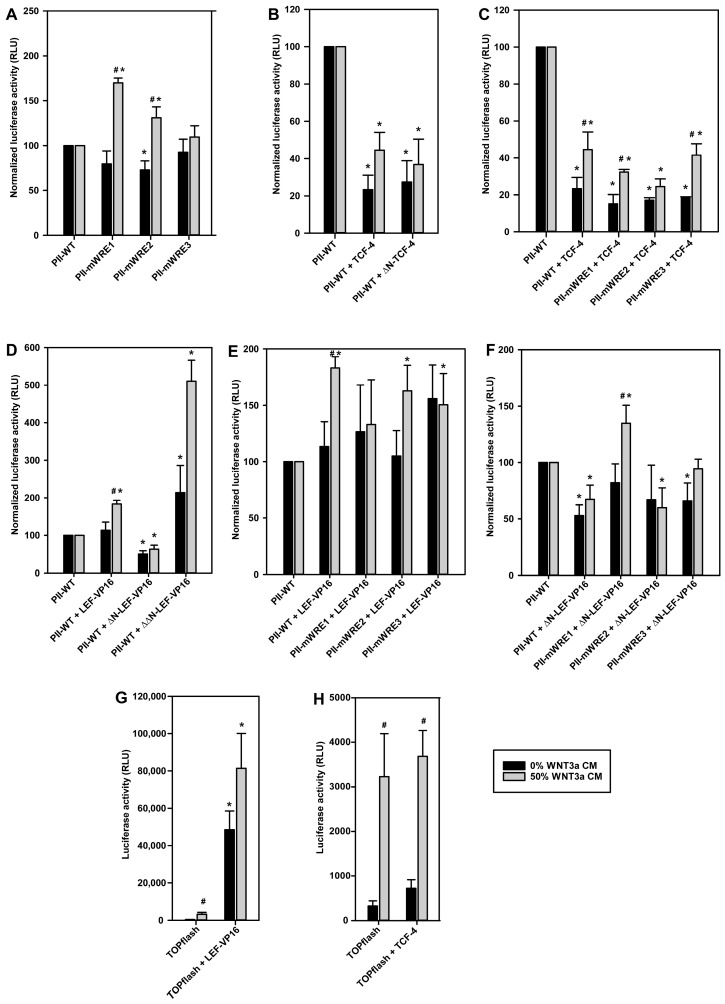
Functional consequences of TCF-4 or LEF-1 binding to WREs in the aromatase promoter I.3/II region. 3T3-L1 preadipocytes were transfected with wildtype aromatase promoter I.3/II reporter constructs pGL3-PII-522 (PII-WT), or derived constructs with inactivating mutations in WRE1 (PII-mWRE1), WRE2 (PII-mWRE2), or WRE3 (PII-mWRE3). Alternatively, the TOPflash Wnt reporter vector was used. For co-transfection vectors containing one of the following, TCF-4 or LEF-1 constructs were used as indicated: pCMV4-FLAG containing full-length TCF-4 (TCF-4) or TCF-4 lacking the N-terminal β-catenin binding domain (ΔN-TCF-4); or pCS2+ containing full-length LEF-1 (LEF-VP16), LEF-1 lacking the N-terminal β-catenin binding domain (ΔN-LEF-VP16) or LEF-1 lacking the β-catenin binding domain, and in addition the context-dependent regulatory domain (ΔΔN-LEF-VP16), which were all fused to the VP16 transactivation domain. As controls, empty pCMV4-FLAG or pCS2+ were used (indications PII-WT or TOPflash in the panels). After 24 h, cells were stimulated in serum-free medium with 10 µM forskolin ± 50% WNT3a CM. Cells were lysed 16 h later, and luciferase activities were measured. For better comparison of effects, the results were normalized to the activities of the respective controls (PII-WT), separately for untreated and WNT3a-treated conditions, respectively, in (**A**–**F**). (**A**) Mutations in WRE1 or WRE2 led to an up to 70% increase in luciferase activities after WNT3a incubation, but tended to be inhibitory in controls. (**B**) Inhibitory effects of TCF-4 and ΔN-TCF-4. (**C**) TCF-4 effects were independent of single WRE mutations. (**D**) Differential effects of LEF-1-VP16 constructs without or with N-terminal deletions. (**E**) WRE1 mutation abolished the significant stimulatory effect of full-length LEF-1-VP16. (**F**) The inhibitory effect of ΔN-LEF-VP16 depended on an intact WRE1. (**G**) LEF-1-VP16 overexpression increased WNT3a stimulation-dependent and -independent reporter gene activities. (**H**) TCF-4 overexpression did not significantly affect luciferase activities. All data are means ± SE of at least 3 experiments. Significant differences to the corresponding controls (PII-WT or TOPflash) (*, *p* < 0.05), or to the corresponding values without WNT3a treatment (#, *p* < 0.05), were identified with Student’s *t*-test.

The evidence obtained so far indicated a major role of WRE1-bound transcription factors of the TCF-4/LEF-1 family in the WNT3a-induced inhibition of transcription at the aromatase promoter I.3/II region. The functional relevance of putative WREs was analyzed further in luciferase reporter gene assays, in 3T3-L1 cells transfected with reporter constructs containing wildtype or WRE-mutated promoter sequences. Starting from the plasmid pGL3-PII-522, where luciferase expression is under the control of the aromatase promoter regions I.3 and II, constructs with individually mutated WREs were generated. These mutations were designed so as to preclude TCF/LEF-binding. Mutation in WRE1 or WRE2 increased promoter activity in WNT3a-stimulated cells, which is in agreement with a role of these WREs in transduction of the inhibitory effect of WNT3a on aromatase promoter I.3/II activity (Figure 6A). Interestingly, in the absence of WNT3a, TCF/LEF binding to WRE2 seems to significantly contribute to full forskolin-dependent activation (Figure 6A). This suggests that WNT3a stimulation might switch WRE1 and WRE2 from an activating to an inhibitory mode. Mutation of WRE3 had no effect on firefly luciferase activity.

For an in-depth analysis of their roles, expression plasmids for full-length or N-terminally truncated variants of TCF-4 or LEF-1 were co-transfected with the aromatase promoter I.3/II reporter plasmids. TCF-4, or ΔN-TCF-4, overexpression resulted in significantly decreased firefly luciferase activities in forskolin stimulated cells, both without and with WNT3a treatment (Figure 6B). This inhibition was also observed, when WREs in the aromatase promoter were individually mutated (Figure 6C). In summary, the inhibitory function of TCF-4 is independent from its N-terminal β-catenin binding region, and is mediated by more than a single WRE (i.e., at least two WREs mediate inhibition by TCF-4).

The ChIP experiments suggested that aromatase promoter I.3/II inhibition might be triggered by increased LEF-1 binding to WRE1. We used LEF-1 constructs, fused to the VP16 transactivation domain from Herpes simplex virus. Previous studies have shown that these constructs activate Wnt target gene transcription, independent of β-catenin [28]. In contrast to TCF-4, full-length LEF-1-VP16 overexpression induced a significant increase in aromatase promoter activity in WNT3a-treated, but not in untreated cells (Figure 6D). Deletion of the N-terminal β-catenin binding region (in the construct ΔN-LEF-1-VP16) eliminated this activating effect of LEF-1-VP16, and transformed it into an inhibitory factor, which acts independently from WNT3a-treatment. In contrast, overexpression of the ΔΔN-LEF-1-VP16 construct, with an in addition deleted context-dependent regulatory domain, increased luciferase activity (up to 400%) (Figure 6D). This construct contains the DNA-binding domain of LEF-1 fused to the transactivation domain of VP16. Taken together, this indicates that the context-dependent regulatory domain (which is present in ΔN-LEF-1 but absent in ΔΔN-LEF-1) is responsible for inhibition of reporter gene activity. The LEF-1 part of the ΔN-LEF-1 construct, therefore, should functionally resemble the lower molecular weight variant upregulated in response to Wnt3a treatment (see Figure 4A).

Unlike in cases of TCF-4 overexpression, the effects of LEF-1-VP16 and ΔN-LEF-1-VP16 overexpression under WNT3a treatment depended on a single WRE. Mutation of WRE1 (almost) eliminated the stimulatory action of full-length LEF-1-VP16 on the aromatase reporter gene (Figure 6E), and it (more than) abolished the inhibitory action of ΔN-LEF-1-VP16 (Figure 6F). Taken together, WRE1 is responsible for the antagonistic actions of LEF-1 isoforms.

To evaluate the effects of TCF-4 and LEF-1-VP16 overexpression on an independent reporter system, the function of both proteins was analyzed by co-transfection of 3T3-L1 preadipocytes with the TOPflash reporter vector, where multiple optimized WREs control luciferase expression. As expected, WNT3a stimulation significantly increased luciferase activity (Figure 6G,H). Co-transfection of LEF-1-VP16 massively increased WNT3a-dependent and -independent luciferase activity in transfected 3T3-L1 cells (Figure 6G), whereas TCF-4 overexpression did not further increase the luciferase activity (Figure 6H). These results indicate that, at least in the 3T3-L1 cell model, a truncated isoform of LEF-1 is the critical factor for Wnt signaling.

## 3. Discussion

In triple-negative breast cancers (TNBC), active Wnt signaling [15,16,17] is associated with poor prognosis [29,30]. Furthermore, Wnt signaling in neighboring adipose tissue may lead to cellular de-differentiation and stabilization of a developmental state of breast adipose fibroblasts (BAFs) [31,32]. Therefore, it is assumed that Wnt signals contribute to the desmoplastic reaction in breast cancers. In this respect, we observed that WNT3a-conditioned media induced an increased growth rate of human BAFs. Similar effects were obtained with conditioned media from all TNBC cell lines. In contrast, conditioned media from receptor-positive cell lines induced heterogenous behavior. Whereas the ER-positive MCF-7 cell line and the HER2-positive SK-BR-3 cell line had no significant effects on cell growth, the ER-positive T-47D and BT-474 cell lines stimulated the growth of BAFs. Therefore, we conclude that canonical Wnt signaling induces BAF accumulation, not solely by forced de-differentiation of adipocytes [31,32], but apparently, in addition, directly promotes BAF proliferation, which would intensify the desmoplastic reaction in the microenvironment of TNBC.

Furthermore, clinical trials revealed a reduced relapse-free period in cases of stromal cell accumulation in TNBC [33], whereas stromal accumulation in ER-positive breast cancers predicted better survival [34]. Hence, the size of the stromal compartment has predictive value regarding the long-term outcome in both of these breast cancer types.

The developmental mechanisms underlying the etiology of diverse breast cancer entities have been increasingly elucidated in recent years, and it has become clear that Wnt signaling is massively involved in normal mammary gland development, as well as in oncogenic dysregulation, as reviewed in, e.g., [35]. For ER-positive tumors, effective treatments are well established. On the other hand, their recognized limitations (e.g., development of endocrine resistance) lead to further optimization of therapies [36]. However, the mechanism(s) responsible for the loss of ERα (and estrogen-dependent growth) in TNBC is (are) currently not clear. These tumors rely on other signaling pathways for growth stimulation, e.g., combined Wnt and Met signaling [37]. In addition, there is a massive discrepancy between ER-positive tumors and triple-negative tumors concerning local estrogen metabolism. In ER-positive tumors the intra-tumoral estrogen concentration can be 10-fold higher than the blood concentration of estrogens [38], and in most cases there is a gradient of aromatase expression towards the tumor in the affected breast (reviewed in [1]). In contrast, in triple-negative cancers, aromatase expression is found only in a minority of samples (and surprisingly is associated with strong androgen receptor expression) [19].

With this background we reasoned that factors driving the growth of TNBC might also be involved in the suppression of aromatase expression in these tumors. Therefore, based on its growth-promoting activities discussed above, and its well-established role in breast (tumor) development [15,16,17], we tested WNT3a for its effect on aromatase induction. Indeed, WNT3a-conditioned medium led to a strong inhibition of aromatase activity in human BAFs. This inhibitory effect correlates with a reduction in aromatase mRNA levels of a similar magnitude, which results from a proportionate decrease in the transcription regulated by the cAMP-dependent aromatase promoters I.3 and II. cAMP-mediated transcriptional activation of aromatase is typical in the vicinity of ER-positive breast cancers [39,40]. Moreover, conditioned medium from triple-negative MDA-MB231 (WNT3a-secreting) cells also inhibited forskolin-induced aromatase activity, and promoter I.3/II mediated aromatase gene expression, however in a less potent manner. This could be due to lower WNT3a levels compared to the conditioned medium from overexpressing L-M(TK-) cells, which were selected for their high WNT3a secretion. Besides that, MDA-MB231 cells may express other canonical Wnt ligands, which are more or less strongly expressed in other breast cancer cell lines [21]. In addition, the known secretion of glucocorticoid-dependent aromatase stimulating factors by MDA-MB231 cells [41], acting via promoter I.4 in target cells (BAFs), may partially antagonize WNT3a-mediated inhibition.

Our results suggest that breast cancer-associated aromatase activity, and estrogen production, not only depend on activating factors from different sources [1], but also on the absence of inhibitory signaling molecules, such as WNT3a. Such a bifunctional model of regulation of breast cancer-associated aromatase expression has not yet been clearly described in the literature. However, it should be noted that a limited number of factors inhibiting aromatase induction in BAFs under certain conditions have been reported. Progesterone can act as a physiological antagonist for glucocorticoid-mediated aromatase induction, via promoter I.4 [42]. In addition, pharmacological doses of RU486 [43] or thiazolidinedione drugs [44] have been shown to repress promoter I.4- and I.3/II-mediated aromatase transcription. Furthermore, some cytokines partially (at best 50%) inhibit induction at of these promoters [1]. However, up to now, no physiological factor has been reported that equals the potency of WNT3a in aromatase inhibition observed in this study.

As a consequence of the results discussed above, it can be concluded that the absence of WNT3a-induced signaling (or effective antagonism, for example by non-canonical Wnts [21]) towards BAFs appears to be of crucial importance for aromatase expression in ER-positive breast cancers. By analogy, this should also hold true for other activators of the canonical Wnt pathway. For an estrogen-dependent tumor, in consequence this implies that the secretion of factors leading to activation of promoter I.3/II-mediated aromatase expression in BAFs is not sufficient to secure a constant supply of estrogens for the tumor cells. Thus, this suggests that ER-positive tumors promote a desmoplastic reaction via factors that concomitantly induce aromatase [1,40], whereas triple-negative tumors drive the desmoplastic reaction predominantly via factors that inhibit aromatase induction. Such a mechanism of differential growth factor secretion also may support a facilitated loss of ERα in initially estrogen-dependent (ER-positive) breast cancers, thereby promoting them to develop a typical TNBC signature. So, if Wnt signaling is activated in BAFs, in a tumor micro-environment rich in non-estrogenic growth factors, the resulting estrogen starvation would favor the growth of cells relying on other growth factors, which would reciprocally make the ER dispensable.

Signals that induce BAFs to secrete WNT proteins, in addition to tumor cells, could lead to some basal autocrine Wnt signaling [45]. Nuclear accumulation of β-catenin is dependent on an active canonical Wnt signaling pathway [6], and was observed in cells irrespective of treatment with WNT3a-conditioned media. This suggests that aromatase expression in the vicinity of breast tumors is controlled by a rather labile signaling environment, where Wnt signaling above a critical threshold will result in a switch-off of estrogen responsiveness/aromatase expression.

Experiments with GSK-3β inhibitors indicated that activation of canonical Wnt signaling is involved in the suppression of aromatase induction in BAFs. It could lead to silencing of aromatase transcription mediated by the promoter region I.3/II via any (combination) of three in silico identified putative Wnt responsive elements (WREs), in this promoter region. Surprisingly, WNT3a treatment, and associated signaling, did not result, as expected, in enhanced association of β-catenin to the WREs, when analyzed by ChIP. In contrast, both in reporter gene assays, and in ChIP experiments, the decisive step for the WNT3a effect on aromatase induction was a switch in WRE1 occupancy from TCF-4 to LEF-1.

Western blots with nuclear extracts from controls and WNT3a-treated cells revealed a change of the expression patterns of TCF-4 and LEF-1, specifically of a WNT3a-induced increase in the levels of an alternative, lower molecular weight LEF-1 variant. The increased expression of the small LEF-1 variant was accompanied by a similar reduction in the amount of the larger variant.

DNA binding assays, with immunoprecipitated nuclear transcription factors, proved that each of the putative WREs can be bound by TCF-4 or LEF-1 in vitro. Unexpectedly, in immunoprecipitates from WNT3a-treated cells, binding to WRE1 and WRE2 was apparently lost. This effect could be traced back to the antibodies used for these immunoprecipitations, which were directed against the N-termini of the proteins, and therefore are not able to bind N-terminally truncated variants. The endogenous full-length TCF-4 or LEF-1 proteins from WNT3a-treated cells seem to lack sufficient ability to bind to WRE1 and WRE2, and preferentially bind to WRE3. In light of the ChIP results, this strongly suggests that Wnt signaling induces preferential binding of an N-terminally truncated LEF-1 variant to WRE1.

Taken together, the findings discussed so far do not perfectly fit to a direct role of canonical Wnt components in the suppression of aromatase induction [6,7,8,9]. Therefore, we systematically analyzed the role of WREs, and various variants of TCF-4 and LEF-1, in reporter gene assays. The emphasis was on N-terminally truncated variants, because these are known for potential antagonistic activities, in comparison to the full-length proteins [46]. Western blot results indicated a WNT3a-induced switch from the full-length LEF-1 isoform to a shorter isoform, which must be truncated N-terminally (because the antibodies used for Western blotting bind more C-terminal regions of their targets than the antibodies discussed in the preceding paragraph). Therefore, N-terminally deleted variants of TCF-4 and LEF-1 were tested for their effects on aromatase promoter I.3/II. Overexpression of full-length LEF-1 resulted in promoter activation, whereas overexpression of ΔN-LEF-1 suppressed luciferase reporter gene activity, both via WRE1. This was the only combination of full-length/truncated factors with a WRE that revealed a switch of the mode of action.

How does alteration of the LEF-1 isoforms produce that switch? Here, comparison of ΔN-LEF-1 with a further truncated variant, ΔΔN-LEF-1, which had lost the β-catenin binding domain, together with the context-dependent regulatory domain, is instructive. The latter domain is crucial for transducin-like enhancer of split (TLE) repressor binding [25,47,48,49]. The inhibitory effects on gene expression of LEF-1 are lost if the association of TLE together with histone deacetylases (HDACs) [50] is lost. The VP16-fusion proteins were used in order to make this effect visible. Consistent with this, the WNT3a-induced suppression of aromatase activity was partially abolished by HDAC inhibition. Therefore, inhibitory HDAC activity, which is very often associated with TLE, cooperates with inhibitory Wnt signaling on the aromatase promoter I.3/II region in BAFs.

In contrast to LEF-1, both the full-length and an N-terminally truncated variant of TCF-4 suppress aromatase promoter I.3/II-dependent reporter gene activity. TCF-4 lost WRE binding ability upon WNT3a treatment of 3T3-L1 cells, or BAFs, in the immunoprecipitation in vitro binding assay (WRE1 and WRE2), and in the ChIP experiments (at least WRE1). Furthermore, using the TOPflash Wnt reporter system, overexpression of TCF-4 does not increase luciferase activity, whereas LEF-1-VP16 does. Taken together, TCF-4 function must be modified in a WNT3a-dependent way, in both 3T3-L1 cells and BAFs.

**Figure 7:**
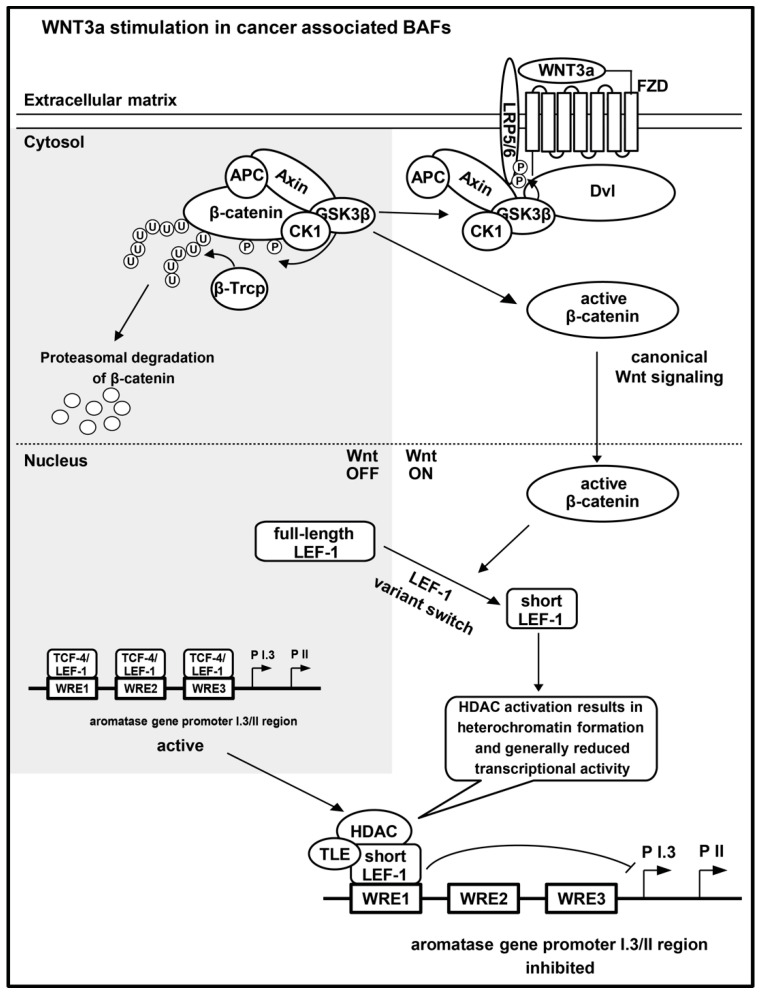
Model for key events in WNT3a-initiated suppression of aromatase expression in BAFs. WNT3a binds Frizzled (FZD) [51], in association with the co-receptor low-density lipoprotein receptor-related protein 5 or 6 (LRP5/6) [52]. In consequence, the β-catenin degradation complex (axin, adenomatous polyposis coli (APC), glycogen synthase kinase 3-β (GSK-3β) and casein kinase 1 (CK1)) is inactivated. GSK-3β and CK1, together with axin and APC, translocate to dishevelled (Dvl) at the membrane, and phosphorylate (P) LRP5/6 [53], but no longer β-catenin. Only phosphorylated β-catenin is ubiquitinylated (U) by E3 ubiquitin ligase β-Trcp (β-Trcp), leading to proteasomal degradation [54]. In canonical Wnt signaling, unphosphorylated β-catenin accumulates and translocates into the nucleus. In forskolin stimulated BAFs, the Wnt-responsive elements (WREs) within the aromatase promoter I.3/II region bind full-length TCF-4 or LEF-1 proteins. WNT3a stimulation of BAFs induces a switch in WRE occupancy: increasing amounts of a nuclear short LEF-1 variant lead to its binding to WRE1. Aromatase promoter I.3/II activation and aromatase expression may be inhibited via heterochromatin formation, involving transducin-like enhancer of split (TLE) and histone deacetylases (HDACs), associated with the short LEF-1 variant. The figure is based on information from some reviews [6,7,8,9].

Finally, the still open question is, “How is this switch from TCF-4 to LEF-1 mediated?” We assume that WNT3a-induced signaling will affect not only aromatase expression in BAFs, but will also induce further changes. In this respect, Wnt signaling was shown to regulate differential expression of LEF-1 and a dominant-negative N-terminally shortened (dnLEF-1) variant. Activation of the Wnt pathway was shown to trigger the switching from promoter 1 utilization (full-length) in the *LEF1* gene, to promoter 2 activation (dnLEF-1) [46]. Although we could not directly verify the identity of the N-terminally shortened LEF-1, and the ΔN-LEF-1 or dnLEF-1 (both lacking the β-catenin binding domain), or the way in which the shortened variant is generated. Our data fit into a unifying model for the mechanism underlying WNT3a-triggered suppression of aromatase expression in BAFs (Figure 7). (1) Canonical Wnt signaling does not directly “activate” the aromatase promoter I.3/II, but instead either induces a promoter switch in the *LEF1* gene, leading to accumulation of dnLEF-1, or induces processing of full-length LEF-1 to the shortened variant. (2) TCF-4 binding to certain WREs (among them WRE1 and WRE2) must be blocked by an unknown, WNT3a-dependent, mechanism, in preadipocyte-like cells (3T3-L1 and BAFs), or may alternatively be outcompeted by the large amount of the short LEF-1 variant. (3) The short LEF-1 variant occupies WRE1 in the promoter I.3/II region. (4) The short LEF-1 variant recruits TLE/HDAC to silence the aromatase promoter.

## 4. Materials and Methods

All chemicals used were of analytical or cell culture grades. All oligonucleotides were from Metabion (Planegg/Steinkirchen, Germany).

### 4.1. Cells and Cell Culture

The 3T3-L1 cells and breast cancer cell lines were obtained from the ATCC (Manassas, VA, USA). The molecular classifications and the culture media for the breast cancer cell lines are summarized in Table 1. Several of these cell lines are known to express various Wnt ligands [21], MDA-MB231 cells are known to secrete WNT3a [18]. The 3T3-L1 cells were cultured in Dulbecco’s Modified Eagle’s Medium (DMEM) (Sigma, Taufkirchen, Germany) containing 10% (*v*/*v*) fetal bovine serum (FBS) (Sera Plus, PAN-Biotech, Aidenbach, Germany) and 40 µg/mL gentamicin. Furthermore, L-M(TK-) cells (parental and WNT3a expressing) were cultured in DMEM containing 10% (*v*/*v*) FBS, 40 µg/mL gentamicin, and 100 µg/mL G418, before being used for production of conditioned media. The production of high titers of WNT3a protein by these cells was verified previously [55].

Human BAFs were isolated from adipose tissue of healthy patients undergoing cosmetic breast reduction surgery. The study was conducted in accordance with the Declaration of Helsinki, and patients gave informed consent according to a protocol approved by the ethics committee of the Jena University Hospital (Ref.-Nr. 4285-12/14). BAFs were isolated and cultured in medium 199, containing 10% (*v*/*v*) FBS and 40 µg/mL gentamicin, as described previously [56]. Confluent primary human BAFs, resembling almost exclusively preadipocytes, were subcultured only once.

All cultured cells were maintained at 37 °C in a humidified atmosphere; 5% CO_2_ and 95% air content were used for all cells and media, except for 3T3-L1 preadipocytes, where the atmosphere contained 7.5% CO_2_ and 92.5% air, during culture in serum-containing growth medium.

If not indicated otherwise, all treatments of cells with stimulators or inhibitors were performed for 24 h, in serum-free medium, consisting of DMEM and Ham’s F12 medium at a ratio of 3:1 (without phenol red and with 7.5 mM HEPES, pH 7.2), which was supplemented with gentamicin (40 μg/mL), transferrin (2 μg/mL), pantothenate (17 μM), biotin (1 μM), and insulin (1 nM) [56]. A general activation of aromatase promoter I.3 and II was obtained by forskolin (10 µM; Cayman Chemicals, Ann Arbor, MI, USA). Furthermore, cells were treated with L-M(TK-)WNT3a or breast cancer cell line conditioned medium (CM), in the concentrations indicated. The conditioned media were collected under serum-free conditions (serum-free medium as used for BAFs) after 3 days conditioning time, and stored at 4 °C after centrifugation. Intracellular processes were inhibited by the broad-spectrum histone deacetylase inhibitor panobinostat (LBH589, IC50 = 5–20 nM, Selleck Chemicals, Houston, TX, USA), and GSK-3β inhibitors lithium chloride (IC50 = 2 mM) and BIO (6-bromoindirubin-3′-oxime, IC50 = 5 nM, Merck, Darmstadt, Germany). When appropriate, DMSO and ethanol solvent controls were carried out in parallel.

### 4.2. Viability Assay with Fluorescein Diacetate

The fluorescein diacetate (FDA) test was carried out as described previously [57]. Two days after passaging of BAFs into 24-well plates (average 5000 cells/cm^2^), the cells were stimulated by up to 50% conditioned media. The conditioned media were mixed with medium 199, containing 10% (*v*/*v*) FBS and 40 µg/mL gentamicin, to ensure a basal growth rate. Stimulations were repeated after 2, 4, and 6 days. On day 8, the FDA test was carried out. After 90 min FDA incubation under cell culture conditions, fluorescence was measured (excitation 480 nm; emission 525 nm, cut off 495 nm) on a SpectraMax M2 plate reader (Molecular Devices, Sunnyvale, CA, USA). All conditions were tested in triplicate or quadruplicate per experiment.

### 4.3. Quantification of mRNA Expression

mRNA expression was quantified by real-time PCR (qPCR). BAF or breast cancer cell mRNA was isolated using the RNeasy Mini Kit (Qiagen, Hilden, Germany) with DNAse digestion. Subsequently, cDNA was synthesized with the High-Capacity cDNA Archive Kit (Applied Biosystems, Darmstadt, Germany), using random hexameric primers. WNT1 and WNT3A expression were analyzed by quantitative real-time PCR (qPCR), in a StepOnePlus cycler (Applied Biosystems), using GoTaq qPCR Master Mix (Promega, Mannheim, Germany), and the following primers (Gene symbol, forward primer, reverse primer): *WNT1*, 5′-GGCAAGATCGTCAACCGAG-3′, 5′-GTCACACGTGCAGGATTCGAT-3′; *WNT3A*, 5′-TTTGGTGGGATGGTGTCTCG-3′, 5′-ACCAGCATGTCTTCACCTCG-3′; and *GAPDH*, 5′-AGCCACATCGCTCAGACAC-3′, 5′-GCCCAATACGACCAAATCC-3′. After 2 min denaturation, 40 cycles of denaturation (15 s), annealing (30 s) and elongation (30 s), were performed. The identities of the products were determined by sequencing and melt-curve analysis. Full-length aromatase mRNA expression and utilization of promoters I.3 and II, respectively, was analyzed by qPCR, as described in detail previously by Wilde et al. [27], using the Universal Probe Library system (Roche, Mannheim, Germany). All conditions were tested in triplicate per experiment. Evaluation of qPCR results was performed by the ΔΔCT-method [58].

### 4.4. Aromatase Activity Testing

The in vivo evaluation of aromatase activity in BAFs was performed using the tritium water release assay, in 24-well plates. The method was carried out as described previously [56,59]. After 18 h in 500 µL serum-free stimulation medium, 1 µCi/well (80 nM) [1β-^3^H]androstenedione (PerkinElmer, Rodgau, Germany) was added as a substrate for the aromatase enzyme 6 h before the incubation was terminated. Aromatase activity was given as pmol androstenedione used per 6 h and mg protein. All conditions were tested in triplicate per experiment.

### 4.5. Preparation of Soluble Nuclear Extracts

The preparation of soluble nuclear extracts was based on a method published by Wilde et al. [27]. The protein concentration was quantified by the Bradford method [60].

### 4.6. Immunoprecipitation-Based DNA-Binding Assay

Soluble nuclear extract proteins (50 µg) were pre-incubated with 20 µL pre-cleared protein G-Sepharose 4 Fast Flow (GE Healthcare, Freiburg, Germany), in soluble nuclear extract buffer, at 4 °C, in a rotator, to eliminate proteins binding nonspecifically to protein G. After centrifugation of the pre-incubated samples (20 s, 12,000× *g*, 4 °C), the supernatants were transferred into new tubes and incubated with antibodies for 24 h, at 4 °C, under constant rotation. The antibodies were, mouse anti-TCF-4 (L40C3) (directed against a peptide around Glu81 of human TCF-4; Cell Signaling Technology, Frankfurt, Germany) or mouse anti-LEF-1 (2D12) (directed against amino acids 1–85 of human LEF-1; nanoTools, Teningen, Germany). After antibody incubation, 20 µL of pre-cleared protein G-Sepharose 4 Fast Flow was added and incubated for 4 h, at 4 °C, under constant rotation. For final isolation of TCF-4 or LEF-1 immunoprecipitates, respectively, the samples were washed three times in a three-fold volume of DNA-binding buffer C (20 mM HEPES pH 7.9, 1 mM EDTA, 1 mM EGTA, 1 mM DTT, 1 mM PMSF), with centrifugation after each step (20 s, 12,000× *g*, 4 °C). The final immunoprecipitates were resuspended in 8 µL buffer C. The DNA-binding reaction was a modification of the sample preparation protocol for electrophoretic mobility shift assays described by Taylor et al. [61]. Immunoprecipitates in buffer C (8 µL) were mixed with 1.7 µL 10-fold binding buffer (500 mM Tris/HCl pH 7.5, 1 M NaCl, 1 mM EDTA, 50 mM β-mercaptoethanol). For normal binding reactions, the premix was added to 2 µL Cy5-labeled double-stranded oligonucleotides (25 pmol/µL; WRE1: 5′-GAGTCACTTTGAATTCAAT-3′, WRE2: 5′-ACTTACTATTTTGATCAAAAAAGTCATT-3′, WRE3: 5′-CTTTTTGTTTTGAAATTGATTTGGCTTCA-3′, only sense sequences given) and 5.3 µL water. For binding reactions in the presence of competitor, 8 µL immunoprecipitate, 2 µL fluorescence-labeled double-stranded oligonucleotides, and 5.3 µL unlabeled competitor double-stranded oligonucleotides (250 pmol/µL; same sequences like fluorescence-labeled oligonucleotides) were mixed. After incubation for 30 min with rotation, at room temperature in the dark, the samples were washed three times in a three-fold sample volume of wash buffer (50% buffer C, 10% 10-fold binding buffer, 40% water), followed by a 20 s centrifugation at 12,000× *g* at room temperature. Finally, the oligonucleotide bound immunoprecipitates were resuspended in 17 µL wash buffer, and transferred to a well of a 96-well plate for fluorescence measurement (excitation 600 nm; emission 670 nm, cut-off 630 nm). As a control, unspecific binding of fluorescent oligonucleotides to protein G-Sepharose 4 Fast Flow beads treated as described above, in the absence of antibodies, was analyzed, resulting in negligible fluorescence signals. All conditions were tested in triplicate per experiment.

### 4.7. Western Blotting

Soluble nuclear extracts (100 µg) of 3T3-L1 cells or BAFs were precipitated with trichloroacetic acid (TCA) and separated on 10% SDS-polyacrylamide gels. Proteins were transferred onto PVDF membranes using semi-dry blotting at 0.8 mA/cm^2^ for 40 min. After blocking in WP-T buffer (10 mM Tris/HCl pH 7.5, 100 mM NaCl, 0.1% (*v*/*v*) Tween 20) with 5% (*w*/*v*) skimmed milk powder, the membrane was incubated overnight with the primary antibodies (1:1000 each): mouse anti-β-catenin (clone 14, 610154, BD Biosciences, Heidelberg, Germany), rabbit anti-TCF-4 (directed against amino acids 486–610 of human TCF-4; H125, sc-13027, Santa Cruz Biotechnology, Heidelberg, Germany), or mouse anti-Lamin A/C (clone 14, 612163, BD Biosciences). Rabbit anti-LEF-1-292 was generated by sequential immunization of a rabbit with purified recombinant LEF-1 (amino acids 1–292) protein [62,63]. After washing in WP-T buffer and blocking in WP-T buffer with 5% (*w*/*v*) milk powder again, the appropriate HRP-conjugated secondary antibodies were added (1:10,000 goat anti-rabbit, 1:5000 goat anti-mouse, Santa Cruz). Proteins were detected using enhanced chemiluminescence.

### 4.8. Plasmid Mutagenesis

Wnt response elements (WREs) were mutated (mWRE) in a pre-existing plasmid, containing firefly luciferase under the control of aromatase promoter region I.3 and II (pGL3-PII-522 WT) [27], by use of the Phusion site-directed mutagenesis protocol (New England Biolabs, Frankfurt, Germany). Primers: mWRE1 (forward) 5′-GTGAGTCACTcgcgATTCAATAGACAAACTGATGGAAGGC-3′, mWRE1 (reverse) 5′-TCAGGCCATCTCTAGTGAC-3′; mWRE2 (forward) 5′-cgcgAAAAGTCATTTTGGTCAAAAAGG-3′, mWRE2 (reverse) 5′-cgcgAATAGTAAGTTTCTACAGTAAGAAC-3′; mWRE3 (forward) 5′-TGTTTTGAAAcgcgTTTGGCTTCAAGGGAAGAAGATTG-3′, mWRE3 (reverse) 5′-AAAAGGCAATCTCCCAAC-3′ (lowercase indicates mutated nucleotide positions). All constructs were verified by sequencing.

### 4.9. Transfection and Luciferase Reporter Gene Assays

Half confluent 3T3-L1 preadipocytes, in 24-well plates, were transfected using Roti-Fect Plus (Carl Roth, Karlsruhe, Germany) for liposome-mediated uptake of DNA, according to the manufacturer’s instructions. The cells were stimulated 24 h later. To quantify promoter activities, the firefly luciferase, containing pGL3-Basic plasmids with wildtype or mutated aromatase promoter I.3/II (pGL3-PII-522-WT, pGL3-PII-522-mWRE1, pGL3-PII-522-mWRE2, pGL3-PII-522-mWRE3, each 800 ng/well), or TOPflash plasmid ([64], kindly provided by Dr. Bert Vogelstein), expressing luciferase under the control of optimized synthetic WREs (150 ng/well), were used. When indicated, cells were co-transfected with plasmids containing either full-length or truncated variants of human TCF-4 or murine LEF-1, respectively (each 400 ng/well): pCMV-FLAG-TCF-4 (596 amino acid protein encoded by GenBank sequence Y11306.2) or pCMV-FLAG-ΔN-TCF-4 (amino acids 32–596 of the aforementioned protein, lacking only the β-catenin binding domain) [65]. Alternatively, pCS2+-LEF-1-VP16 (397 amino acid protein encoded by RefSeq sequence NM_010703.4), pCS2+-ΔN-LEF-1-VP16 (amino acids 57–397, lacking only the β-catenin binding domain), or pCS2+-ΔΔN-LEF-1-VP16 (amino acids 265–397, lacking β-catenin binding domain and context dependent regulatory domain), all fused to the VP16 activation domain as internal control, were used, which were described previously [28,65]. All conditions were tested in triplicate per experiment.

### 4.10. Chromatin Immunoprecipitation (ChIP)

The chromatin immunoprecipitation protocol is a modified version of that published by Weinmann and Farnham [66,67]. BAFs from four 10 cm dishes per condition were used. For protein G-based immunoprecipitation, 1 µg/reaction rabbit anti-LEF-1 292, rabbit anti-TCF-4X (H125), or mouse anti-β-catenin (clone 14) antibodies were used. Primer set 1 amplifies the region containing the three putative WREs in aromatase promoter I.3/II: 5′-TGAAGTCACTAGAGATGGCCTG-3′ (forward), 5′-GCTCATTCCAGAGGTGGAGTC-3′ (reverse). Primer set 2 amplifies a region containing putative WRE2 and WRE3, but not WRE1: 5′-GGCTCTGAGAAGACCTCAACG-3′ (forward), 5′-GTAGAGTGACGTGCATTCCCA-3′ (reverse). PCR was performed using Paq5000 DNA polymerase (Agilent Technologies, Santa Clara, CA, USA), and PCR-products were analyzed on 12% (*w*/*v*) polyacrylamide gels stained with ethidium bromide, as described previously [27].

### 4.11. Statistical Analyses

Statistical analyses of all experiments, and creation of diagrams, were performed with the SigmaPlot 13 or 14 software (Systat, Erkrath, Germany). Data are presented as means ± SEM or using box plots, where appropriate. Initially, normal distribution of values was tested by the Shapiro–Wilk method. Normally distributed values were compared to another group by the two-tailed Student’s *t*-test (if not explicitly indicated otherwise in text/legends). In the case of non-normally distributed values, two groups were compared by the Mann–Whitney rank sum test, if indicated. For all tests, the significance criterion *p* < 0.05 was used. All numbers of replications (n) in figure legends refer to biological replicates.

## 5. Conclusions

Canonical Wnt signaling toward BAFs can induce change in a breast tumor environment in two ways: it can initiate/enhance the desmoplastic reaction, and thus increase the amount of altered stroma; and it can suppress local estrogen production in the BAFs. Therefore, breast tumors, which secrete Wnt ligands may cut themselves off from a sufficient estrogen supply for growth promotion. Lacking estrogen signaling consequently will make ERα dispensable, and thus supports development into a hormone receptor-negative tumor.

## Figures and Tables

**Table 1:** Breast cancer cell lines used in the study.

Cell Line	Molecular Classification	ER	PR	HER2	WNT	Growth Medium
MDA-MB468	TNA	-	-	-	[21]	DMEM/10% (*v*/*v*) FBS
BT-20	TNA	-	-	-	[21]	DMEM/10% (*v*/*v*) FBS
HCC-1143	TNA	-	-	-		DMEM/20% (*v*/*v*) FBS
MDA-MB231	TNB	-	-	-	[18]	DMEM/10% (*v*/*v*) FBS
MCF-7	LA	+	+	-	[21]	DMEM/10% (*v*/*v*) FBS
T-47D	LA	+	+	-	[21]	RPMI/10% (*v*/*v*) FBS
BT-474	LB	+	+	+	[21]	RPMI/10% (*v*/*v*) FBS
SK-BR-3	H	-	-	+		RPMI/10% (*v*/*v*) FBS

Notes: The breast cancer cell lines were chosen to represent different molecular classifications, as suggested by Neve et al. [22] and Dai et al. [23]: triple negative A (TNA), triple negative B (TNB), luminal A (LA), luminal B (LB), and receptor tyrosine-protein kinase erbB-2 (HER2) positive (H), + or − indicate expression of ERα (ER), PR, or HER2; WNT, expression of WNT genes is described in the reference given; growth medium, indicates the medium used for propagation of the individual cell line.

## Data Availability

The data that support the findings of this study are available from the corresponding author upon reasonable request.

## References

[1] Chen D., Reierstad S., Lu M., Lin Z., Ishikawa H., Bulun S.E. (2009). Regulation of breast cancer-associated aromatase promoters. Cancer Lett..

[2] Simpson E.R., Clyne C., Rubin G., Boon W.C., Robertson K., Britt K., Speed C., Jones M. (2002). Aromatase--a brief overview. Annu. Rev. Physiol..

[3] Simpson E.R., Mahendroo M.S., Means G.D., Kilgore M.W., Hinshelwood M.M., Graham-Lorence S., Amarneh B., Ito Y., Fisher C.R., Michael M.D. (1994). Aromatase cytochrome P450, the enzyme responsible for estrogen biosynthesis. Endocr. Rev..

[4] Santen R.J., Brodie H., Simpson E.R., Siiteri P.K., Brodie A. (2009). History of aromatase: Saga of an important biological mediator and therapeutic target. Endocr. Rev..

[5] Johnston S.R., Dowsett M. (2003). Aromatase inhibitors for breast cancer: Lessons from the laboratory. Nat. Rev. Cancer.

[6] MacDonald B.T., Tamai K., He X. (2009). Wnt/β-catenin signaling: Components, mechanisms, and diseases. Dev. Cell.

[7] Lien W.-H., Fuchs E. (2014). Wnt some lose some: Transcriptional governance of stem cells by Wnt/β-catenin signaling. Genes. Dev..

[8] Niehrs C. (2012). The complex world of WNT receptor signalling. Nat. Rev. Mol. Cell Biol..

[9] Anastas J.N., Moon R.T. (2013). WNT signalling pathways as therapeutic targets in cancer. Nat. Rev. Cancer.

[10] Polakis P. (2007). The many ways of Wnt in cancer. Curr. Opin. Genet. Dev..

[11] Cadigan K.M., Waterman M.L. (2012). TCF/LEFs and Wnt signaling in the nucleus. Cold Spring Harb. Perspect. Biol..

[12] Hovanes K., Li T.W., Munguia J.E., Truong T., Milovanovic T., Lawrence Marsh J. (2001). β-Catenin-sensitive isoforms of lymphoid enhancer factor-1 are selectively expressed in colon cancer. Nat. Genet..

[13] van de Wetering M., Castrop J., Korinek V., Clevers H. (1996). Extensive alternative splicing and dual promoter usage generate Tcf-protein isoforms with differential transcription control properties. Mol. Cell. Biol..

[14] Duval A., Rolland S., Tubacher E., Bui H., Thomas G., Hamelin R. (2000). The human T-cell transcription factor-4 gene: Structure, extensive characterization of alternative splicings, and mutational analysis in colorectal cancer cell lines. Cancer Res..

[15] Geyer F.C., Lacroix-Triki M., Savage K., Arnedos M., Lambros M.B., MacKay A., Natrajan R., Reis-Filho J.S. (2011). β-Catenin pathway activation in breast cancer is associated with triple-negative phenotype but not with CTNNB1 mutation. Mod. Pathol..

[16] Khramtsov A.I., Khramtsova G.F., Tretiakova M., Huo D., Olopade O.I., Goss K.H. (2010). Wnt/β-catenin pathway activation is enriched in basal-like breast cancers and predicts poor outcome. Am. J. Pathol..

[17] Lamb R., Ablett M.P., Spence K., Landberg G., Sims A.H., Clarke R.B. (2013). Wnt pathway activity in breast cancer sub-types and stem-like cells. PLoS ONE.

[18] Bochet L., Lehuédé C., Dauvillier S., Wang Y.Y., Dirat B., Laurent V., Dray C., Guiet R., Maridonneau-Parini I., Le Gonidec S. (2013). Adipocyte-derived fibroblasts promote tumor progression and contribute to the desmoplastic reaction in breast cancer. Cancer Res..

[19] McNamara K.M., Oguro S., Omata F., Kikuchi K., Guestini F., Suzuki K., Yang Y., Abe E., Hirakawa H., Brown K.A. (2017). The presence and impact of estrogen metabolism on the biology of triple-negative breast cancer. Breast Cancer Res. Treat..

[20] Stapp A.D., Gómez B.I., Gifford C.A., Hallford D.M., Hernandez Gifford J.A. (2014). Canonical WNT signaling inhibits follicle stimulating hormone mediated steroidogenesis in primary cultures of rat granulosa cells. PLoS ONE.

[21] Benhaj K., Akcali K.C., Ozturk M. (2006). Redundant expression of canonical Wnt ligands in human breast cancer cell lines. Oncol. Rep..

[22] Neve R.M., Chin K., Fridlyand J., Yeh J., Baehner F.L., Fevr T., Clark L., Bayani N., Coppe J.P., Tong F. (2006). A collection of breast cancer cell lines for the study of functionally distinct cancer subtypes. Cancer Cell.

[23] Dai X., Cheng H., Bai Z., Li J. (2017). Breast Cancer Cell Line Classification and Its Relevance with Breast Tumor Subtyping. J. Cancer.

[24] Willert K., Brown J.D., Danenberg E., Duncan A.W., Weissman I.L., Reya T., Yates J.R., Nusse R. (2003). Wnt proteins are lipid-modified and can act as stem cell growth factors. Nature.

[25] Billin A.N., Thirlwell H., Ayer D.E. (2000). β-Catenin-histone deacetylase interactions regulate the transition of LEF1 from a transcriptional repressor to an activator. Mol. Cell. Biol..

[26] Mosimann C., Hausmann G., Basler K. (2009). β-Catenin hits chromatin: Regulation of Wnt target gene activation. Nat. Rev. Mol. Cell Biol..

[27] Wilde J., Erdmann M., Mertens M., Eiselt G., Schmidt M. (2013). Aromatase activity induction in human adipose fibroblasts by retinoic acids via retinoic acid receptor α. J. Mol. Endocrinol..

[28] Aoki M., Hecht A., Kruse U., Kemler R., Vogt P.K. (1999). Nuclear endpoint of Wnt signaling: Neoplastic transformation induced by transactivating lymphoid-enhancing factor 1. Proc. Natl. Acad. Sci. USA.

[29] López-Knowles E., Zardawi S.J., McNeil C.M., Millar E.K.A., Crea P., Musgrove E.A., Sutherland R.L., O’Toole S.A. (2010). Cytoplasmic localization of β-catenin is a marker of poor outcome in breast cancer patients. Cancer Epidemiol. Biomark. Prev..

[30] Lin S.-Y., Xia W., Wang J.C., Kwong K.Y., Spohn B., Wen Y., Pestell R.G., Hung M.-C. (2000). β-Catenin, a novel prognostic marker for breast cancer: Its roles in cyclin D1 expression and cancer progression. Proc. Natl. Acad. Sci. USA.

[31] Gustafson B., Smith U. (2010). Activation of canonical wingless-type MMTV integration site family (Wnt) signaling in mature adipocytes increases β-catenin levels and leads to cell dedifferentiation and insulin resistance. J. Biol. Chem..

[32] Christodoulides C., Lagathu C., Sethi J.K., Vidal-Puig A. (2009). Adipogenesis and WNT signalling. Trends Endocrinol. Metab..

[33] Moorman A.M., Vink R., Heijmans H.J., van der Palen J., Kouwenhoven E.A. (2012). The prognostic value of tumour-stroma ratio in triple-negative breast cancer. Eur. J. Surg. Oncol..

[34] Downey C.L., Simpkins S.A., White J., Holliday D.L., Jones J.L., Jordan L.B., Kulka J., Pollock S., Rajan S.S., Thygesen H.H. (2014). The prognostic significance of tumour-stroma ratio in oestrogen receptor-positive breast cancer. Br. J. Cancer.

[35] Rangel M.C., Bertolette D., Castro N.P., Klauzinska M., Cuttitta F., Salomon D.S. (2016). Developmental signaling pathways regulating mammary stem cells and contributing to the etiology of triple-negative breast cancer. Breast Cancer Res. Treat..

[36] Ali S., Coombes R.C. (2002). Endocrine-responsive breast cancer and strategies for combating resistance. Nat. Rev. Cancer.

[37] Holland J.D., Gyorffy B., Vogel R., Eckert K., Valenti G., Fang L., Lohneis P., Elezkurtaj S., Ziebold U., Birchmeier W. (2013). Combined Wnt/β-catenin, Met, and CXCL12/CXCR4 signals characterize basal breast cancer and predict disease outcome. Cell Rep..

[38] van Landeghem A.A., Poortman J., Nabuurs M., Thijssen J.H. (1985). Endogenous concentration and subcellular distribution of estrogens in normal and malignant human breast tissue. Cancer Res..

[39] Zhao Y., Agarwal V.R., Mendelson C.R., Simpson E.R. (1996). Estrogen biosynthesis proximal to a breast tumor is stimulated by PGE2 via cyclic AMP, leading to activation of promoter II of the CYP19 (aromatase) gene. Endocrinology.

[40] Zhou J., Gurates B., Yang S., Sebastian S., Bulun S.E. (2001). Malignant breast epithelial cells stimulate aromatase expression via promoter II in human adipose fibroblasts: An epithelial-stromal interaction in breast tumors mediated by CCAAT/enhancer binding protein β. Cancer Res..

[41] Schmidt M., Löffler G. (1994). The human breast cancer cell line MDA-MB231 produces an aromatase stimulating activity. Eur. J. Cell Biol..

[42] Schmidt M., Renner C., Löffler G. (1998). Progesterone inhibits glucocorticoid-dependent aromatase induction in human adipose fibroblasts. J. Endocrinol..

[43] Schmidt M., Löffler G. (1997). RU486 is a potent inhibitor of aromatase induction in human breast adipose tissue stromal cells. J. Steroid Biochem. Mol. Biol..

[44] Rubin G.L., Duong J.H., Clyne C.D., Speed C.J., Murata Y., Gong C., Simpson E.R. (2002). Ligands for the peroxisomal proliferator-activated receptor gamma and the retinoid X receptor inhibit aromatase cytochrome P450 (CYP19) expression mediated by promoter II in human breast adipose. Endocrinology.

[45] Augsten M. (2014). Cancer-associated fibroblasts as another polarized cell type of the tumor microenvironment. Front. Oncol..

[46] Li T.W.-H., Ting J.-H.T., Yokoyama N.N., Bernstein A., van de Wetering M., Waterman M.L. (2006). Wnt activation and alternative promoter repression of LEF1 in colon cancer. Mol. Cell. Biol..

[47] Arce L., Yokoyama N.N., Waterman M.L. (2006). Diversity of LEF//TCF action in development and disease. Oncogene.

[48] Brantjes H., Roose J., van De Wetering M., Clevers H. (2001). All Tcf HMG box transcription factors interact with Groucho-related co-repressors. Nucleic Acids Res..

[49] Daniels D.L., Weis W.I. (2005). β-Catenin directly displaces Groucho/TLE repressors from Tcf/Lef in Wnt-mediated transcription activation. Nat. Struct. Mol. Biol..

[50] Arce L., Pate K.T., Waterman M.L. (2009). Groucho binds two conserved regions of LEF-1 for HDAC-dependent repression. BMC Cancer.

[51] Logan C.Y., Nusse R. (2004). The Wnt signaling pathway in development and disease. Annu. Rev. Cell Dev. Biol..

[52] He X., Semenov M., Tamai K., Zeng X. (2004). LDL receptor-related proteins 5 and 6 in Wnt/β-catenin signaling: Arrows point the way. Development.

[53] Liu X., Rubin J.S., Kimmel A.R. (2005). Rapid, Wnt-induced changes in GSK3β associations that regulate β-catenin stabilization are mediated by Gα proteins. Curr. Biol..

[54] Kimelman D., Xu W. (2006). β-Catenin destruction complex: Insights and questions from a structural perspective. Oncogene.

[55] Ye Z., Mittag S., Schmidt M., Simm A., Horstkorte R., Huber O. (2019). Wnt Glycation Inhibits Canonical Signaling. Cells.

[56] Schmidt M., Löffler G. (1998). Induction of aromatase activity in human adipose tissue stromal cells by extracellular nucleotides. Eur. J. Biochem..

[57] Albring K.F., Weidemuller J., Mittag S., Weiske J., Friedrich K., Geroni M.C., Lombardi P., Huber O. (2013). Berberine acts as a natural inhibitor of Wnt/β-catenin signaling--identification of more active 13-arylalkyl derivatives. Biofactors.

[58] Pfaffl M.W. (2001). A new mathematical model for relative quantification in real-time RT-PCR. Nucleic Acids Res..

[59] Ackerman G.E., Smith M.E., Mendelson C.R., Macdonald P.C., Simpson E.R. (1981). Aromatization of androstenedione by human adipose tissue stromal cells in monolayer culture. J. Clin. Endocrinol. Metab..

[60] Bradford M.M. (1976). A rapid and sensitive method for the quantitation of microgram quantities of protein utilizing the principle of protein-dye binding. Anal. Biochem..

[61] Taylor J.D., Ackroyd A.J., Halford S.E., Kneale G.G. (1994). The gel shift assay for the analysis of DNA-protein interactions. DNA–Protein Interactions: Principles and Protocol.

[62] Huber O., Huber-Wunderlich M. (2000). Recombinant proteins. J. Chromatogr. Libr..

[63] Cribier B., Worret W.I., Braun-Falco M., Peltre B., Langbein L., Schweizer J. (2006). Expression patterns of hair and epithelial keratins and transcription factors HOXC13, LEF1, and β-catenin in a malignant pilomatricoma: A histological and immunohistochemical study. J. Cutan. Pathol..

[64] Korinek V., Barker N., Morin P.J., van Wichen D., de Weger R., Kinzler K.W., Vogelstein B., Clevers H. (1997). Constitutive transcriptional activation by a β-catenin-Tcf complex in APC-/-colon carcinoma. Science.

[65] Mittag S., Valenta T., Weiske J., Bloch L., Klingel S., Gradl D., Wetzel F., Chen Y., Petersen I., Basler K. (2016). A novel role for the tumour suppressor Nitrilase1 modulating the Wnt/β-catenin signalling pathway. Cell Discov..

[66] Weinmann A.S., Farnham P.J. (2002). Identification of unknown target genes of human transcription factors using chromatin immunoprecipitation. Methods.

[67] Weiske J., Huber O. (2006). The histidine triad protein Hint1 triggers apoptosis independent of its enzymatic activity. J. Biol. Chem..

